# Supplementary material for: The effect of obesity on the outcome of thoracic endovascular aortic repair: a systematic review and meta-analysis
Source: PeerJ. 2024 Apr 19;12:e17246. doi: 10.7717/peerj.17246 (PMC11034506; doi:10.7717/peerj.17246)
Supplement: Supplemental Information 2 [file peerj-12-17246-s002.docx]

Supplementary File

Supplementary Table 1. Baseline characteristics of enrolled cohort studies

| **Author** | **Year** | **Area** | **Diseases** | **Study**  **Design** | **Sample Size** | **Age** | **Male** | **Exposure** | **Comparator** |
| --- | --- | --- | --- | --- | --- | --- | --- | --- | --- |
| Isaac N. Naazie | 2022 | United States and Canada | Thoracic aortic aneurysm or type B dissections | Prospective cohort study | 3419 | 69.0±12.1 | 1469 | BMI≥30 | 30>BMI≥18.5 |
| Anne-Sophie C. Romijn | 2023 | America | BTAI | Retrospective cohort study | 2022 | female vs. male: 46 years [IQR 30, 62] vs. 39 years [IQR 28, 56] | 1499 | BMI＞30 | BMI≤30 |
| Ottavia Borghese | 2023 | France | Pararenal/Thoracoabdominal aortic aneurysm and Dissection | Prospective cohort study | 195 | 69.6 ±11.2 | 135 | BMI≥30 | BMI＜30 |
| Ali Khoynezhad | 2007 | America | DTAA, TBAD, Penetrating thoracic ulcers, Traumatic aortic transections. | Prospective cohort study | 153 | 71±13.7 | 94 | BMI＞30 | BMI≤30 |
| Mengye Niu | 2023 | China | TBAD | Retrospective cohort study | 684 | 52.7±6.1 | 514 | BMI＞28.06 | BMI≤24.22 |
| Zhengbiao Zha | 2022 | China | TBAD | Retrospective cohort study | 445 | 53.0±3.2 | 240 | BMI≥30 | BMI＜24 |
| Nan Lu | 2020 | China | TBAD | Retrospective cohort study | 269 | 55.5 ± 9.9 | 226 | BMI≥30 | BMI＜25 |
| Dariusz Janczak | 2019 | Wroclaw | TAA, ATBAD | Retrospective cohort study | 51 | 66.3 | 34 | BMI＞30 | BMI＜25 |

DTAA = descend thoracic aortic aneurysm; TBAD = type B aortic dissection; ATBAD = acute type B aortic dissection; TAA = thoracic aortic aneurysm; BMI =body mass index; BTAI = blunt thoracic aortic injury; IQR = interquartile range

Supplementary Table 2. Baseline characteristics of studies conducted case-control analysis nested in cohort studies

| **Author** | **Year** | **Area** | **Diseases** | **Study Design** | **Sample Size** | **Age** | **Male** | **Case** | **Control** |
| --- | --- | --- | --- | --- | --- | --- | --- | --- | --- |
| Mengye Niu | 2023 | China | TBAD | Retrospective cohort study | 684 | 52.7±6.1 | 514 | poor  outcome | no complications |
| Jason Zakko | 2014 | America | Patients  receiving TEVER | Retrospective cohort study | 355 | 64.2±16.3 | 270 | failure of (P-TEVAR) | success of (P-TEVAR) |
| Xiuping An | 2021 | China | TBAD and renal artery involvement | Retrospective cohort study | 256 | 52.8 ± 9.8 | 217 | AKI | non-AKI |

TBAD = type B aortic dissection; TBAD = type B aortic dissection; TEVAR = thoracic endovascular aortic repair; AKI = acute kidney diseases.

Supplementary Table 3. Quality assessment of Cohort studies by Newcastle–Ottawa Scale

| Included Studies | Selection | | | |  | Comparability | |  | Outcome | | | Total Scores |
| --- | --- | --- | --- | --- | --- | --- | --- | --- | --- | --- | --- | --- |
|  | A | B | C | D |  | A1 | B1 |  | A2 | B2 | C2 |  |
| Khoynezhad 2007 | 1 | 1 | 1 | 0 |  | 1 | 1 |  | 1 | 1 | 1 | 8 |
| Zakko 2014 | 1 | 1 | 1 | 0 |  | 1 | 1 |  | 1 | 1 | 1 | 8 |
| Janczak 2019 | 1 | 1 | 1 | 0 |  | 1 | 1 |  | 1 | 1 | 1 | 8 |
| Lu 2020 | 1 | 1 | 1 | 0 |  | 0 | 0 |  | 1 | 1 | 1 | 6 |
| Naazie 2022 | 1 | 1 | 1 | 0 |  | 1 | 1 |  | 1 | 1 | 1 | 8 |
| An 2021 | 1 | 1 | 1 | 0 |  | 1 | 1 |  | 1 | 1 | 1 | 8 |
| Zha 2022 | 1 | 1 | 1 | 0 |  | 1 | 1 |  | 1 | 1 | 1 | 8 |
| Niu 2023 | 1 | 1 | 1 | 0 |  | 0 | 0 |  | 1 | 1 | 1 | 6 |
| Romijn 2023 | 1 | 1 | 1 | 0 |  | 1 | 1 |  | 1 | 1 | 1 | 8 |
| Borghese 2023 | 1 | 1 | 1 | 0 |  | 1 | 1 |  | 1 | 1 | 1 | 8 |

A: Representativeness of exposed cohort. B: Representativeness of unexposed cohort. C: Ascertainment of exposure (If the exposure data was obtained from prescription database or medical record). D: Outcome was not present at start. A1: Important factor (If adjusted for the age, a point was assigned.) B1: Additional factor (If adjusted for any other additional factors.) A2: Assessment of outcome. B2: Exposure Follow-up for outcomes. C2: Rate of follow-up

Supplementary Table 4. Associations between obesity and some complications after thoracic endovascular aortic repair.

| Study | Complication | Odds ratio | 95% confidence interval | p-value |
| --- | --- | --- | --- | --- |
| Dariusz 2019 | neurological complication | 0.13 | 0.01-2.37 | 0.17 |
| Ottavia 2023 | endoleak | 1.02 | 0.46-2.29 | 0.96 |
|  | wound complication | 0.91 | 0.28-2.96 | 0.88 |
|  | renal failure | 2.98 | 0.92-9.69 | 0.07 |
|  | re-intervention (30-day) | 1.80 | 0.56-5.76 | 0.33 |
|  | re-intervention (90-day) | 0.77 | 0.24-2.45 | 0.66 |
|  | re-intervention (long-term) | 0.44 | 0.16-1.20 | 0.11 |


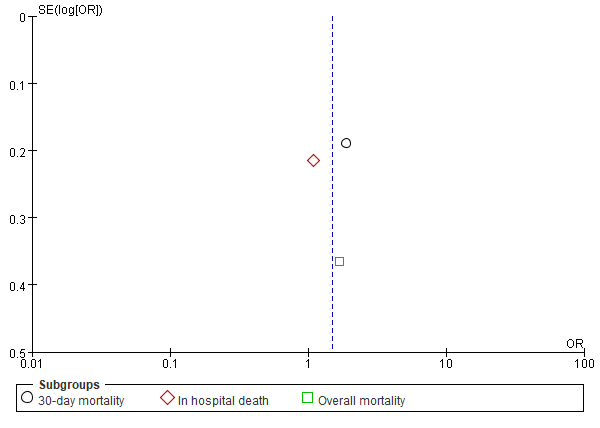


Supplementary Figure 1. The funnel plot for the pooled analysis on postoperative mortality


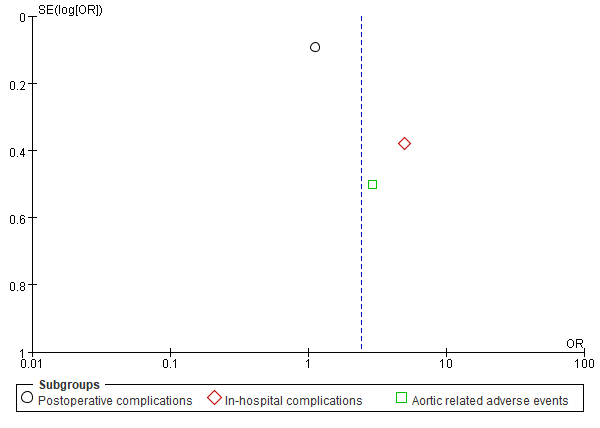


Supplementary Figure 2. The funnel plot for the pooled analysis on overall postoperative complications


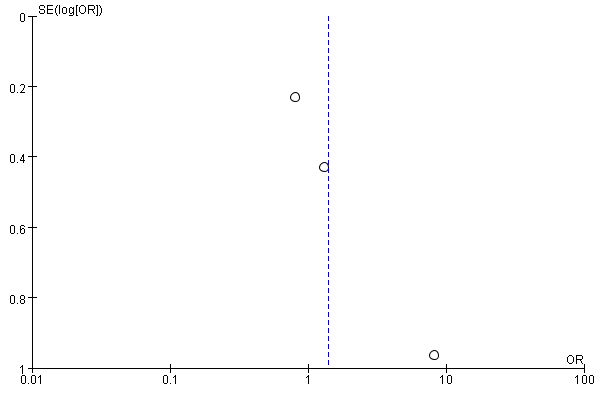


Supplementary Figure 3. The funnel plot for the pooled analysis on postoperative stroke


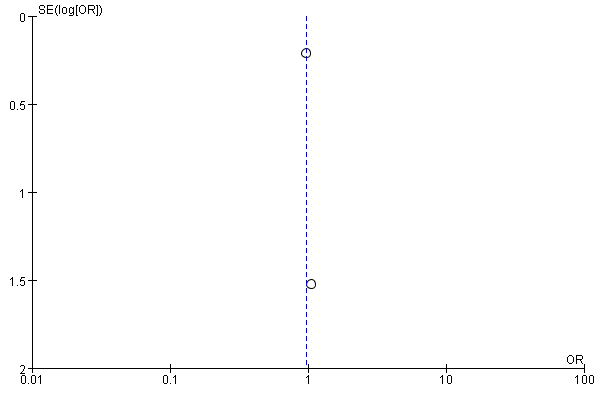


Supplementary Figure 4. The funnel plot for the pooled analysis on postoperative spinal ischemia
